# Signatures in Vibrational and Vibronic Spectra of Benzene Molecular Clusters

**DOI:** 10.1021/acs.jpca.4c08700

**Published:** 2025-04-09

**Authors:** Ricardo Montserrat, Amanda D. Torres, Ricardo R. Oliveira, Alexandre B. Rocha

**Affiliations:** † Chemistry Institute, Federal University of Rio de Janeiro, Rio de Janeiro 21941-909, Brazil; ‡ Instituto Federal do Paraná, Umuarama, Paraná 87507-014, Brazil

## Abstract

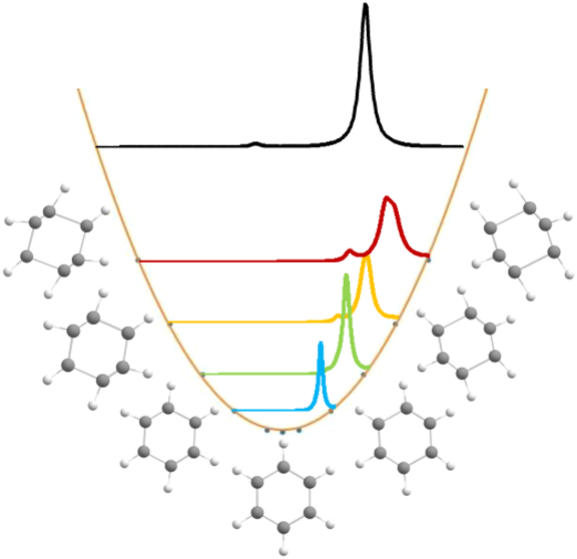

The photoabsorption and infrared spectra (IR) of molecular systems are heavily influenced by aggregation. In the electronic spectra, the vibronic coupling effect is of utmost importance. Although treating both effects simultaneously can be challenging, it is often the only way to explain the experimental spectrum of molecular clusters. In this work, we study IR spectra and the vibronic coupling effect in the electronic photoabsorption spectra in molecular systems composed of benzene (monomer, dimers, and crystal). Photoabsorption spectra were generated using the direct vibronic coupling method at the density functional theory (DFT) level. We also simulated the spectra with the Liouville-Lanczos approach by calculating the electronic transitions along the main inducing modes for two forbidden transitions (^1^A_1g_ → ^1^B_2u_ and ^1^A_1g_ → ^1^B_1u_). DFT was also applied to simulate IR spectra. For the monomer, vibronic coupling was crucial to induce the first and second forbidden transitions. On the other hand, molecular aggregation was sufficient to induce the first and second forbidden transitions in almost all dimers. However, when the vibronic coupling is evaluated for the clusters, the band in the energy range of the ^1^A_1g_ → ^1^B_1u_ transition is affected both by the aggregation itself and the inducing modes. Moreover, some inducing modes drastically change the allowed ^1^A_1g_ → ^1^E_1u_ transition, depending on the dimer under study due to symmetry breaking. In terms of IR spectra, clear signatures are present. For instance, the intensities of the C–H stretching modes decrease as aggregation increases. This work shows that aggregation impacts the band shapes differently in relation to the benzene aggregate structure and the excitation under analysis.

## 1 Introduction

Molecular aggregation strongly impacts the spectroscopic and electronic properties of molecular crystals. This phenomenon can be used to adjust the optical features of organic materials for varying applications. For instance, an active research area on this topic is the development of materials in organic optoelectronics.^1−3^ Therefore, tuning the performance of optoelectronic devices, such as organic light-emitting diodes (OLEDs), organic field-effect transistors (OFETs), and organic solar cells, is achievable through modifications at the molecular organization level.^2,4^ An organic semiconductor (OSC) might undergo many photophysical pathways in these devices, such as absorption, emission, charge separation, singlet fission, triplet–triplet annihilation, and so forth.^5^ Understanding these steps provides means to improve OSC’s effectiveness. Specifically, photoabsorption in the ultraviolet–visible (UV–vis) region, which is strongly dependent on vibronic transitions, is a critical stage. In this respect, progress has been made in understanding the interplay between the aggregate structure and the vibronic coupling.^6−8^

Regarding the influence of the cluster structure in the absorption spectrum, the Kasha exciton model can describe the energetic shifts of excited states in molecular aggregates.^9−11^ Excitons in organic aggregates frequently exhibit small wave function overlap, and excitations are predominantly localized in the molecular units.^5^ Thus, this approximation considers the coulombic intermolecular coupling as the only perturbation that leads to changes in the excited electronic states in H– and J–aggregates. Although the success shown by the Kasha model in explaining the electronic spectra of molecular clusters is remarkable, it does not include vibrational effects and wave function overlap among molecules.^12^ Therefore, if short-range interactions between the molecules are not negligible and vibronic transitions are present, the Kasha model might fail in predicting and explaining the aggregate’s spectrum. However, more sophisticated approaches are available to rationalize the effects of aggregation on absorption spectra today.^13−16^

Planar molecules with aromatic cores (e.g., acenes, naphthalene, and perylene derivatives, among others) constantly are employed as OSCs for developing organic optoelectronic materials.^17−20^ In this context, benzene is a prototype for simulating the behavior of the absorption spectrum upon aggregation. Although it has a higher band gap than the OSCs cited previously (∼3.6 eV),^21,22^ it is a simple aromatic molecule, showing π–π interactions in its aggregates, such as the typical OSCs. To further ensure that benzene is an important molecular prototype for the study of larger molecule spectra, some recent works on the stability and spectroscopic features were performed for polycyclic aromatic hydrocarbons^23,24^ and fullerenes.^25^ The study of spectra simulations using a cluster approach was also performed, but the vibronic coupling was included based only on the monomer vibrational functions.^16,17^ An important conclusion from recent works was the presence of spectroscopic signatures as a function of charge state and molecular aggregation.^16,17,23−25^

The equilibrium geometry of the benzene monomer (gas-phase isolated molecule) belongs to the D_6h_ point group. The electronic ground state is ^1^A_1g_, and the lowest singlet excited states are ^1^B_2u_, ^1^B_1u_, and ^1^E_1u_. The lowest valence transitions involving these states are π → π*excitations. The ^1^A_1g_ → ^1^B_2u_ (4.9 eV)^26^ and ^1^A_1g_ → ^1^B_1u_ (6.19 eV)^26^ transitions are forbidden by the electric dipole approximation. On the other hand, the ^1^A_1g_ → ^1^E_1u_ (6.96 eV)^26^ transition is allowed. Due to vibronic coupling, the lowest excited singlets are weakly accessible through photoabsorption. Also, these transitions show roughly an intensity ratio of 1:10:100.^26^ A thorough study of the absorption features of benzene can be carried out due to the large amount of experimental^27−34^ and theoretical^35−43^ data known for this molecule.

Considering the importance and ubiquity of IR spectra in chemical analysis, it is also of interest to identify possible signatures of aggregation in it.

Thus, this work aims to study spectroscopic signatures in infrared (IR) and vibronic spectra of benzene aggregates and crystal.

## 2 Methods

### 2.1 Molecular Geometries

Molecular geometry optimizations for the monomer and dimer were carried out in Gaussian 16^44^ and Crystal23^45^ software. Also, for the benzene crystal, geometry optimization and frequency computations with frozen lattice constants were performed using experimental cell parameter values only using the Crystal software.^46^ The ωB97X-D/aug-cc-pVTZ level of theory was employed for the dimers in Gaussian. Both dimers used in this work were taken from the crystalline structure of benzene at high pressures (0.7 GPa).^46^ In addition, the B3LYP functional combined with 6–311G(d) basis set by Heyd^47^ and 5–11G(d) basis set by Dovesi^48^ were used in Crystal software for carbon and hydrogen, respectively.

### 2.2 Absorption Spectra

Absorption spectra of selected molecular clusters were calculated using two different approaches based on time-dependent density functional theory (TDDFT). Gaussian 16^44^ was used to obtain vertical excitations at the ωB97X-D/6–31+G(d) level of theory. In addition, the Liouville-Lanczos approach, available in Quantum ESPRESSO software (version 6.4),^49−51^ was used to calculate intensities and energies for the transitions at the PBE/PW level of theory, where PW stands for “plane waves”. The Liouville-Lanczos approach is a powerful method for calculating the entire valence absorption spectrum (including the ionization continuum) within the linear response regime of TDDFT. The method was developed^51^ to avoid the traditional approach to solve the linearized Liouville equation to obtain the spectrum for individual states. It relies on the idea that for large systems the spectrum becomes so dense that it can essentially be represented by a continuous function, i.e., the imaginary part of the dynamic susceptibility, even before the ionization edge. So, it is excellent for dealing with large systems.^52^ Periodic boundary conditions (PBC) were used with cubic cells of 30 Å lattice parameter.

### 2.3 Vibronic Coupling

An investigation of vibronic effects was carried out using the direct vibronic coupling (DVC) method.^53−56^ Briefly, in the DVC method, the total wave function is written as the product of the vibrational and electronic wave functions. The intensity of excitation from the lowest vibrational level of the ground electronic state to the vibrational level *ν* of the excited electronic state *k* can be obtained by the optical oscillator strength (OOS), which is given by
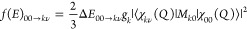
1where the transition dipole moment is
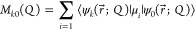
2with ψ_*k*_ an electronic wave function, χ_*kν*_ vibrational wave functions, and μ_*i*_ the dipole moment operator. Also *r⃗* are the electron coordinates and *Q* are the nuclear coordinates.

The square of the transition dipole moment is written as the product of the bracket times its conjugate complex and, using the completeness relation and summing over all vibrational levels of the excited electronic state, the expression can be written as
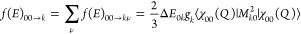
3

Now, the previous dependence on the excited vibrational functions no longer exists, and only the ground state enters the equation, in addition to the dependence on electronic degrees of freedom through the transition moment. Furthermore, the ground state vibration function can be suitably approximated by the harmonic oscillator function for this state (ξ _L_(*Q*_L_)). In addition, the transition dipole moment can be expanded in a power series, and the transition energy is replaced by the vertical energy. After these approximations, the final expression will be
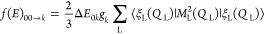
4being
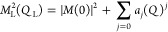
5

The first term (|*M*(0)|^2^) represents the transition dipole moment calculated on the equilibrium geometry, while the summation (∑_*j*=0_*a*_*j*_(*Q*)^*j*^) is in the coefficients *a*_*j*_ obtained from a polynomial fitting on *M*^2^ vs *Q* for a given normal mode. Substituting the harmonic oscillator wave function and integrating the terms, we get:
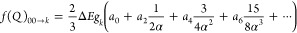
6

Furthermore, a qualitative study of the vibronic coupling was performed using the spectrum obtained with the Liouville-Lanczos approach. In this case, we analyzed the behavior of the spectrum with the molecules subjected to distortions along the inducing modes for selected transitions. For the monomer, after choosing the inducing modes, we calculated the photoabsorption spectra with progressively larger deformation throughout the modes. We generated and compared ten spectra within 1–10% molecular distortions along each mode. For the dimers, we only calculated the spectra for 10% of molecular cluster distortions along the inducing modes since the number of normal modes increases considerably compared to the monomer. It is worth noticing that intensities are not averaged considering the contribution of each mode in this study, as we do in the DVC method. In all cases, the band broadening was simulated using Lorentzian functions.

## 3 Results and Discussion

### 3.1 Simulated Infrared Spectra

**Figure 1:**
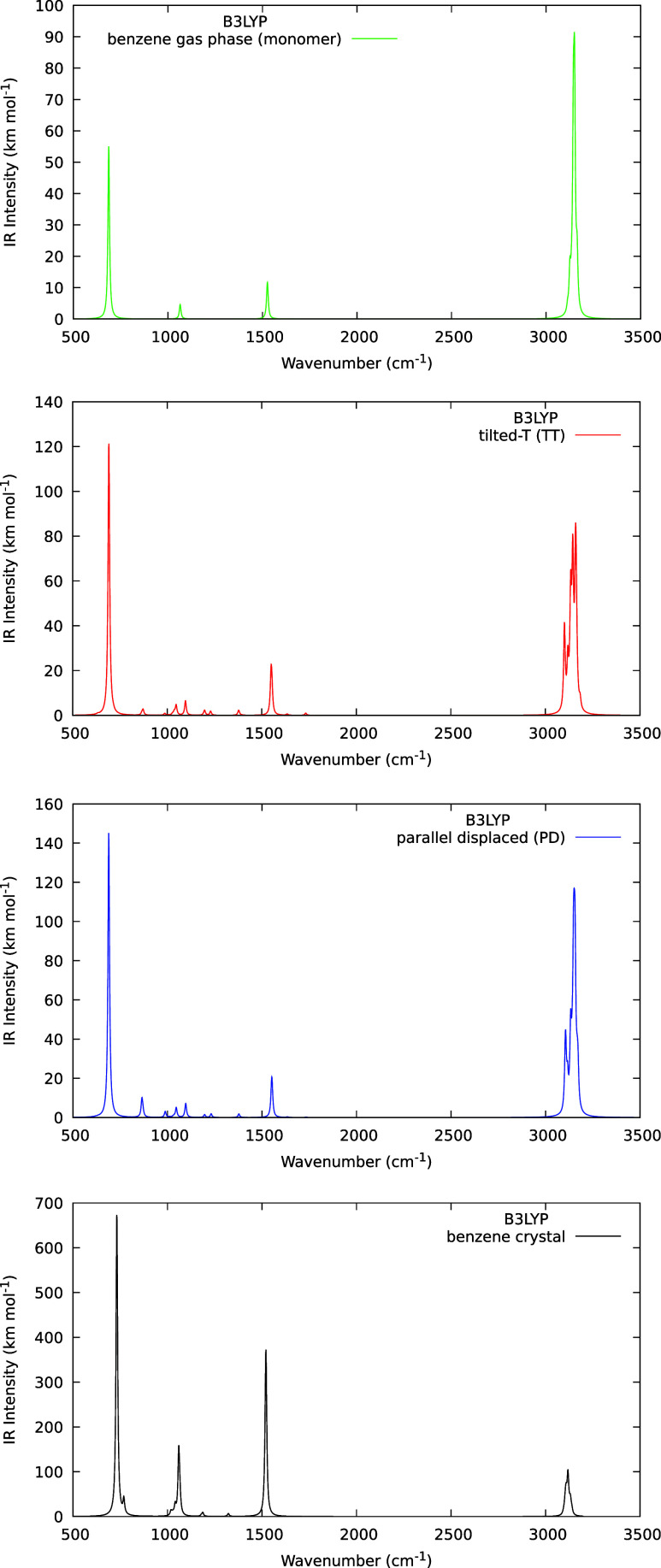
Simulated infrared spectra of benzene monomer at gas phase (first panel), tilted-T dimer (second panel), parallel displaced dimer (third panel), and crystal (solid) phase (fourth panel) obtained with B3LYP functional implemented in Crystal software.

The gas-phase infrared spectrum of benzene is present in Figure 1, first panel, which is in good agreement with the experimental one,^57,58^ i.e., only four bands are present with E_1u_ and A_2u_ symmetries. Concerning dimers spectra (Figure 1, second and third panels), the C–H stretching region (>3000 cm^–1^) presents a more complex band profile due to the symmetry reduction and the most intense band is related to C–C–C bending mode (around 687 cm^–1^). The same occurs in the region between 500 and 1500 cm^–1^ wavenumbers. In the crystalline form, the benzene infrared spectrum (Figure 1, fourth panel) exhibits the lowest-intensity band in the C–H stretching region with good agreement with experimental data.^59^ In comparison with the gas-phase spectrum, the most intense band in the crystal (around 731 cm^–1^) presents a red shift of 45 cm^–1^. From experimental results, this red shift value was estimated at between 17 and 34 cm^–1^,^60^ which is in reasonable agreement with our simulated results due to the lack of anharmonic corrections.

### 3.2 Vibronic Coupling in Benzene Monomer

**Figure 2:**
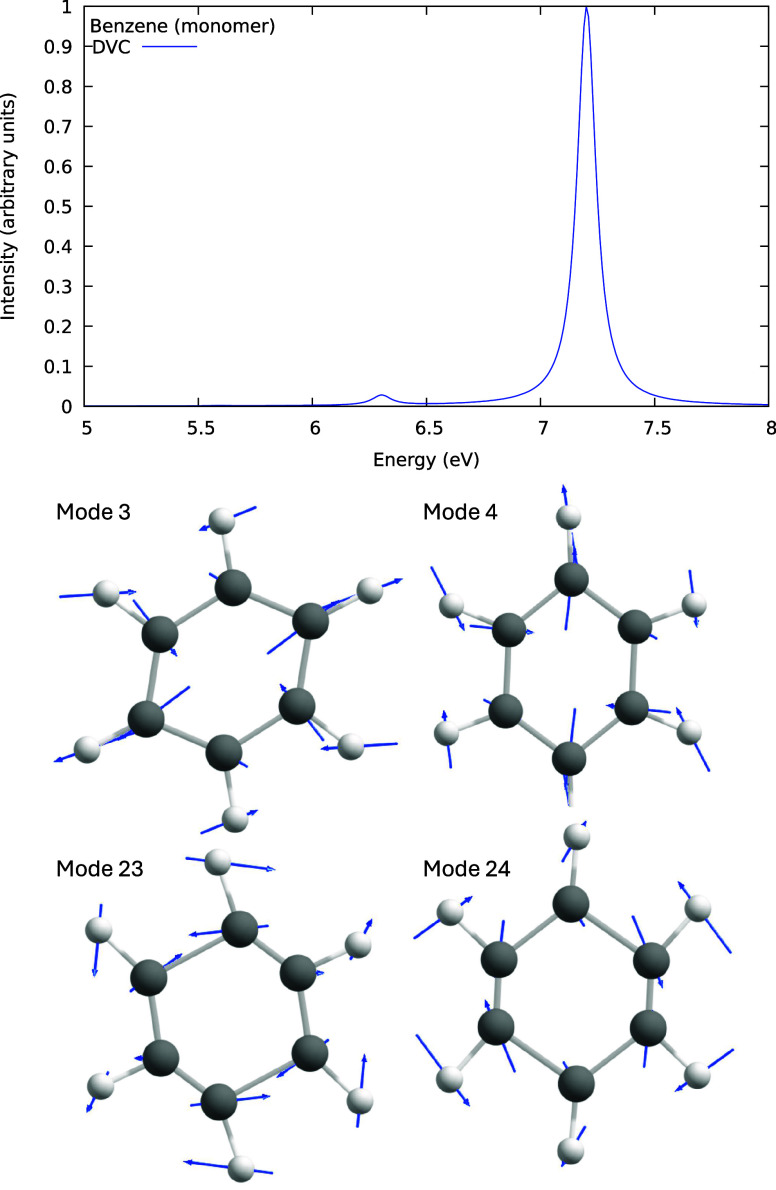
Simulated absorption spectrum of benzene monomer (gas phase) obtained using density functional theory (DFT), specifically the ωB97X-D/6–31+G(d) level of theory (top). The main inducing modes for transition 1 (^1^A_1g_ → ^1^B_2u_) are presented in the middle panel, while those for transition 2 (^1^A_1g_ → ^1^B_1u_) are shown in the bottom panel. The blue arrows on each atom represent the scaled normal mode vectors.

It is well known that the absorption spectrum computed at the equilibrium geometry of benzene fails to reproduce the experimental spectrum due to forbidden transitions activated solely through vibronic coupling. The states involved in these electronic transitions are of symmetry ^1^A_1g_ and ^1^B_2u_ and ^1^A_1g_ and ^1^B_1u_, for the first two bands, respectively.^26^ As benzene belongs to the point group D_6h_, the dipole moment, which is transformed like position vectors (*x*,*y*,*z*), is of symmetry E_1g_ and A_2u_. As previously mentioned, in order to have an allowed vibronic transition, the transition dipole moment must be totally symmetric, i.e., the direct product of the dipole moment, electronic and vibrational functions must result in A_1g_. In other words, all modes with symmetry such that the direct product of the vibrational and electronic irreducible representations yields a totally symmetric representation are expected to induce the vibronic transition. For the first transitions, the inducing modes will be of symmetry:
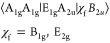
For the second transition, inducing modes will be
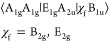
All modes with symmetry B_1g_, B_2g_, and E_2g_, i.e., modes 3, 4, 6, 12, 23, 24, 27, and 28 (shown in Table S1), were considered on the spectra. Results obtained from DVC are shown in Table 1 and Figure 2 (top). The inducing modes are not the most intense ones in the IR spectra of benzene monomers, dimers, and crystals, as we can see in Figure 1.

**Table 1:** Contribution Per Inducing Vibrational Mode to the OOS for Transition 1, ^1^A_1g_ → ^1^B_2u_ (top) and for Transition 2, ^1^A_1g_ → ^1^B_1u_, (Bottom) in C_6_H_6_ Monomer

^1^A_1g_ → ^1^B_2u_	symmetry	OOS	%
Mode 3	E_2g_	5.35 × 10^–3^	49.86
Mode 4	E_2g_	5.27 × 10^–3^	49.12
Mode 17	E_2g_	2.02 × 10^–7^	0.00
Mode 18	E_2g_	5.23 × 10^–7^	0.00
Mode 23	E_2g_	6.28 × 10^–6^	0.06
Mode 24	E_2g_	9.66 × 10^–6^	0.09
Mode 26	E_2g_	4.49 × 10^–5^	0.42
Mode 27	E_2g_	4.72 × 10^–5^	0.44
total		1.07 × 10^–2^	

^1^A_1g_ → ^1^B_1u_	symmetry	OOS	%
Mode 3	E_2g_	3.79 × 10^–3^	1.25
Mode 4	E_2g_	3.22 × 10^–3^	1.07
Mode 6	B_2g_	2.86 × 10^–4^	0.09
Modo 12	B_2g_	1.48 × 10^–3^	0.49
Mode 17	E_2g_	8.08 × 10^–3^	2.67
Mode 18	E_2g_	9.11 × 10^–3^	3.01
Mode 23	E_2g_	1.39 × 10^–1^	45.85
Mode 24	E_2g_	1.38 × 10^–1^	45.53
Mode 26	E_2g_	2.90 × 10^–5^	0.01
Mode 27	E_2g_	7.50 × 10^–5^	0.02
total		3.02 × 10^–1^	

For the first transition (vertical transition energy of 5.59 eV), the most prominent inducing modes are by far Modes 3 and 4, which are ring deformation modes. They change the interatomic distances between two opposite carbon atoms, compressing the ring. It also changes the neighbor hydrogen distance but with a smaller amplitude. The integrated OOS is so small that it cannot be visualized on the spectrum scale in Figure 2 (top). For the second transition (vertical transition energy of 6.31 eV), the primary inducing modes are 23 and 24, which are ring stretch modes. A minimal intensity is present for this transition around the vertical transition energy (see Figure 2, bottom). All of these modes are shown in Figure 2 (bottom). Our results are similar to those obtained with the CASSCF method.^39^ A more detailed discussion of these transitions is presented below.

**Figure 3:**
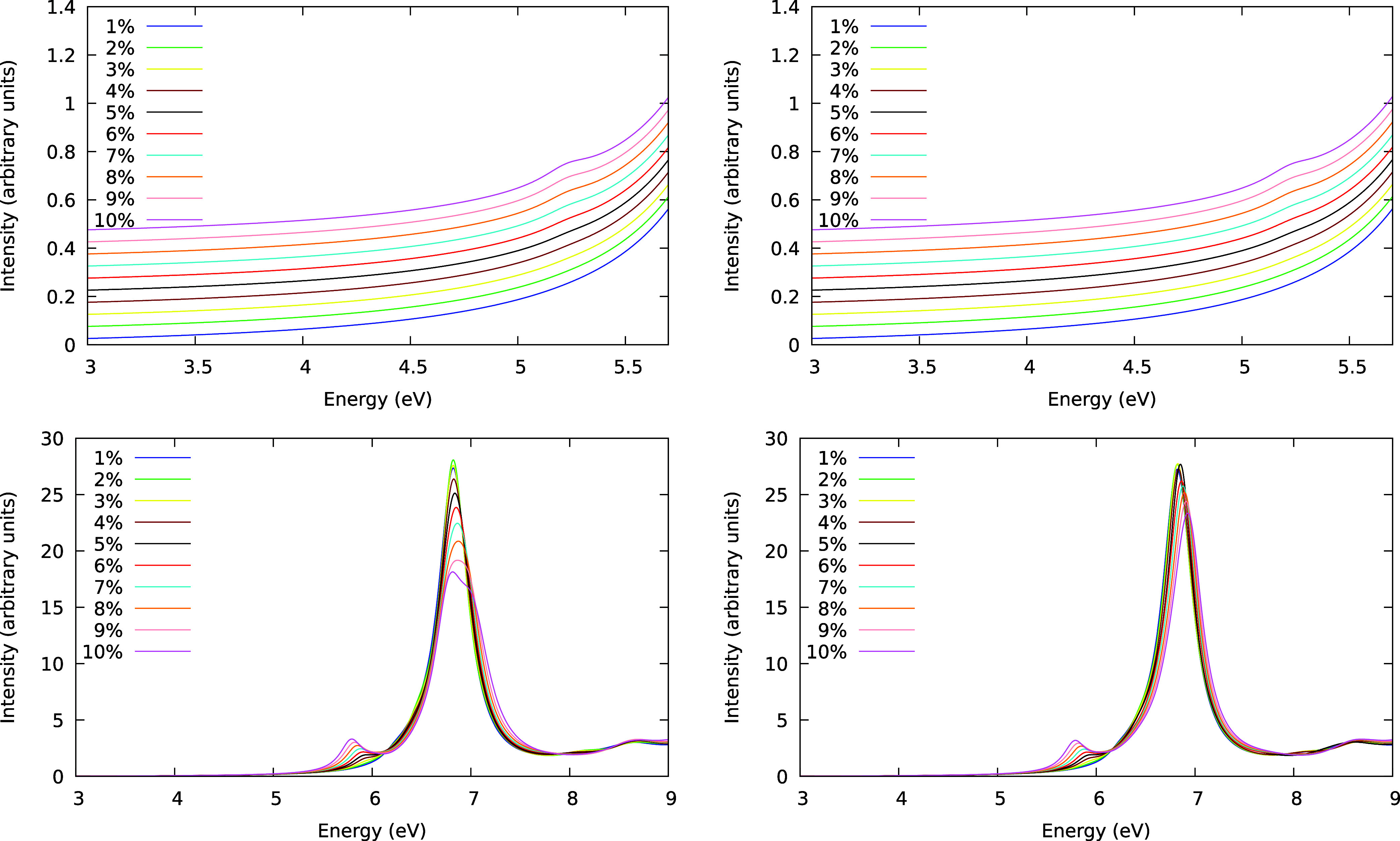
Gas-phase benzene absorption spectra calculated by the Liouville-Lanczos method for molecular geometries distorted along modes 3 (left) and 4 (right) in the ^1^A_1g_ → ^1^B_2u_ energy range (top panel) and along modes 23 (left) and 24 (right) in the ^1^A_1g_ → ^1^B_1u_ energy range (bottom panel). The percentages indicate the molecular deformation along each mode. The spectra are presented on different scales to facilitate a better understanding of the data. The top panel spectra are vertically shifted to enhance data visualization.

The same study was conducted using the Liouville-Lanczos method, but this time, only the most inducing modes were considered. For mode 3 (E_2g_ symmetry), the induced forbidden electronic transition has its experimental band maximum around 4.9 eV. Therefore, Figure 3 (top left panel) displays the region around this energy. When the molecular geometry is only slightly deformed, no peak is observed in this region of the spectrum. However, with increasing deformation along the normal mode until 10% of distortion, a band emerges around 5.2 eV, suggesting that this mode induces the ^1^A_1g_ → ^1^B_2u_ excitation. The small band intensity closely aligns with the experimental results. The other strongly inducing mode for the first electronic transition is mode 4 (E_2g_ symmetry). Figure 3 (top right panel) presents the absorption spectrum analysis along this mode of vibration. The result obtained for this mode is remarkably similar to the one obtained for mode 3 (Figure 3, top right panel). This similarity is anticipated, as these two modes are degenerate (Table 1). For both modes analyzed so far (modes 3 and 4), a blue shift of 0.3 eV occurs compared to the experimental energy for this peak.

The second state accessed via vibronic coupling (^1^B_1u_), mainly induced by modes 23 (E_2g_ symmetry) and 24 (E_2g_ symmetry), was also investigated. Figure 3 (bottom left panel) shows that with increasing distortion along mode 23, a band appears at 5.8 eV. Similarly, this behavior is present in the case of mode 24 (Figure 3, bottom right panel). Although the ^1^A_1g_ → ^1^E_1u_ transition OOS raises in both modes almost identically, surprising trends happen for the allowed ^1^A_1g_ → ^1^E_1u_ transition in each case (6.8 eV). For the analysis involving mode 23, the ^1^A_1g_ → ^1^B_1u_ transition decreases the intensity more than the observed in mode 24. Moreover, for mode 23, the band slowly splits into two separate transitions, i.e., a shoulder rises in the distorted molecule spectrum. The same trend is noticed with DVC, as seen in Table 2. The difference in the energy of the two peaks increases with increasing distortion along the mode.

**Table 2:** Energy Difference between the First and Second Vertical Transition That Compose the Allowed Band along the Normal Mode 23, Obtained with the DVC Method

distortion (%)	OOS (1)	OOS (2)	Δ*E*
0			
1	0.6386	0.6417	0.0067
3	0.6332	0.6278	0.0379
5	0.6273	0.6012	0.0900
7	0.6209	0.5686	0.1574
9	0.6140	0.5335	0.2350

### 3.3 Vibronic Coupling in Benzene Dimers

**Figure 4:**
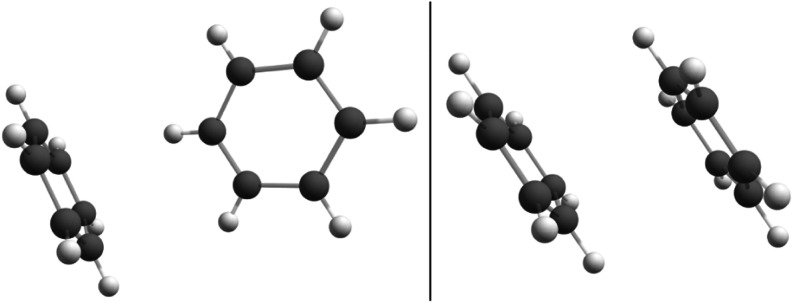
Structures of the tilted-T (left) and parallel displaced (right) benzene dimers.

A comparable study was conducted for two pairs of distinct aggregates of benzene: one exhibiting a parallel displaced (PD) geometry and the other presenting a tilted-T (TT) geometry, both shown in Figure 4. High-level quantum calculations^61,62^ and experimental results^63,64^ indicate that the TT-shaped structure is the global minimum of the gas-phase dimer. However, TDDFT tends to predict the PD dimer as the minimum, with the TT dimer being almost isoenergetic to it.^65^ This discrepancy arises from the poor description of van der Waals interactions by DFT.^62^ Consequently, both structures were considered in this work. As is well known, the resolution for the hydrogen positions is not resolved with prediction in monocrystal X-ray diffraction data. Therefore, these atoms were optimized while the carbon ones were frozen (a restricted optimization). Nevertheless, these structures are considered experimental ones. Parallel to it, these structures were also fully optimized, and these results are compared in the next sections.

#### 3.3.1 Experimental Structures

**Figure 5:**
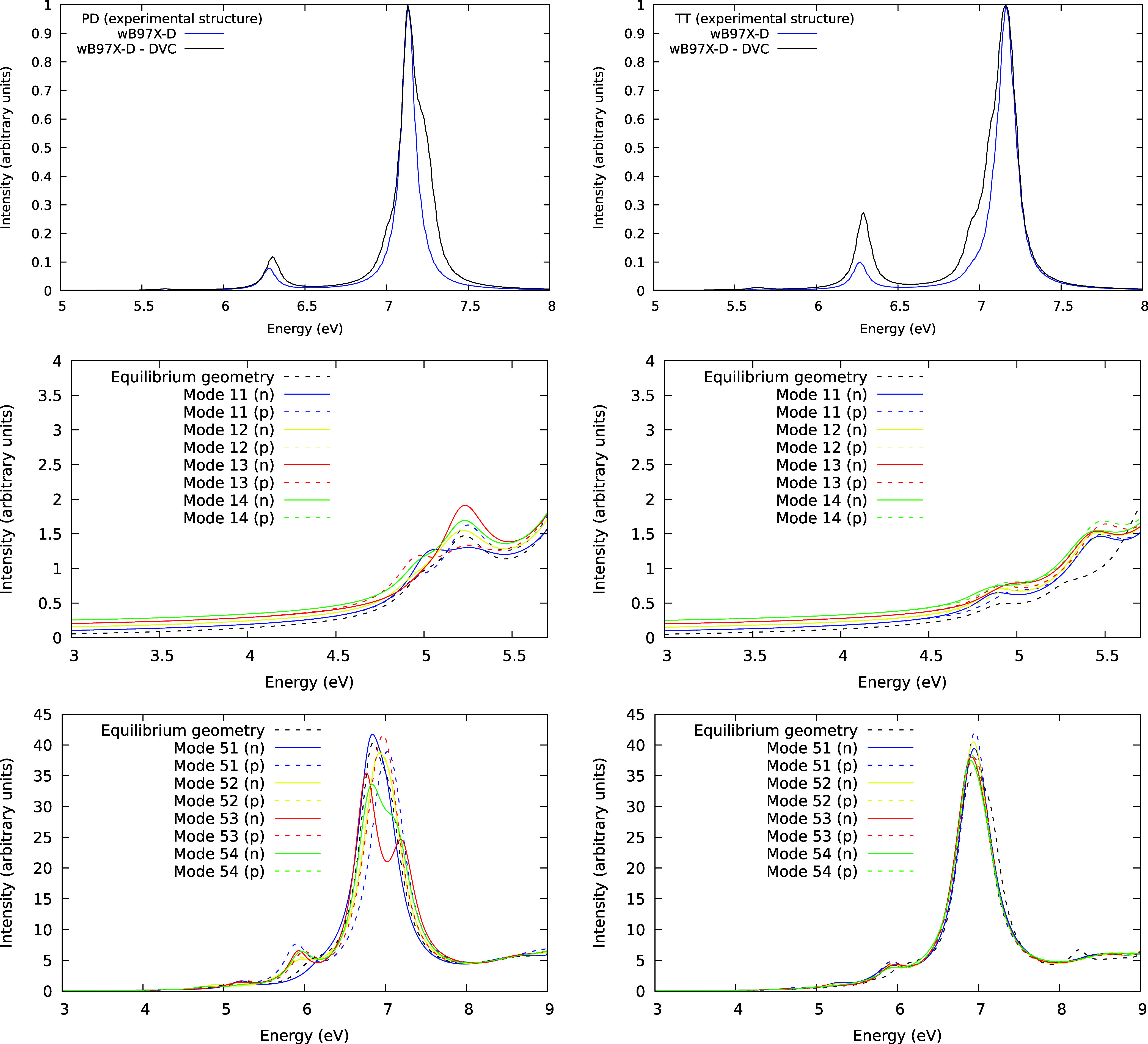
Simulated absorption spectra utilizing the experimental structure for the PD dimer (left) and TT dimer (right) using DVC (top panel, blue line), compared with the spectra on equilibrium geometry (black lines) and Turbo-Laczos (middle and bottom panels) distorted along inducing modes 11, 12, 13, and 14, (middle panel) and 51, 52, 53, and 54, (bottom panel) and dislocated 10% in both directions along the mode, are also shown. The middle panel spectra are vertically shifted for enhanced visualization of the data. These modes were anticipated to induce an excitation in the same energy range as the ^1^A_1g_ → ^1^B_2u_ and ^1^A_1g_ → ^1^B_1u_ respectively.

Only the distortions for most inducing modes from the monomer were considered for the dimers: four-ring deformations and four-ring stretches. The criteria for selecting the dimer modes were the similarity between those modes and the corresponding inducing modes in the monomer in terms of energy and nuclear motion. As expected for the dimers, two doubly degenerate modes were observed for each deformation instead of one, as observed on the monomer. Their simulated spectra using DVC are presented in Figure 5 (top panels). Both the PD and the TT dimers exhibit a shoulder in the energy range between 7.00 and 7.50 eV. However, the shoulder is positioned on the right-hand side in the PD dimer, whereas it is on the left-hand side in the TT dimer. Additionally, for the TT dimer, there was a greater increase in intensity for the low-energy band. This could be attributed to the greater break of symmetry provided by these geometries, as their π system is not frontally interacting due to the “T” cluster shape.

Using the Liouville-Lanczos approach, Figure 5 (middle left panel) shows that for the experimental PD dimer, a relatively prominent band is already present in the equilibrium geometry. The distorted geometries along the inducing modes do not show a significant increase in the OOSs compared to the equilibrium geometry, except for mode 13 (*n*). Mode 11 (*n*) splits the band into two excitations. On the other hand, the inducing modes couple to the electronic transitions to increase the excitation intensities for the experimental TT dimer (Figure 5, middle right panel). Furthermore, it can be seen that two transitions occur at 4.9 and 5.4 eV, approximately. In the case of the second forbidden transition, Figure 5 (bottom left panel) shows that the inducing modes (except mode 51 (*n*)) are crucial to augment the OOSs at about 6 eV for the experimental PD dimer. Only a little shoulder is present for the equilibrium geometry. Curiously, the allowed ^1^A_1g_ → ^1^E_1u_ transition (around 7 eV) is highly affected depending on the distortion imposed on the molecules. For instance, the modes 53 (*n*) and 54 (*n*) break the degeneracy of the ^1^E_1u_ state. For the TT dimer (Figure 5, bottom right panel), all inducing modes increase the intensity of the band at 6 eV. Interestingly, we also observe a small increase in the band’s intensity at 7 eV.

#### 3.3.2 Optimized Structures

**Figure 6:**
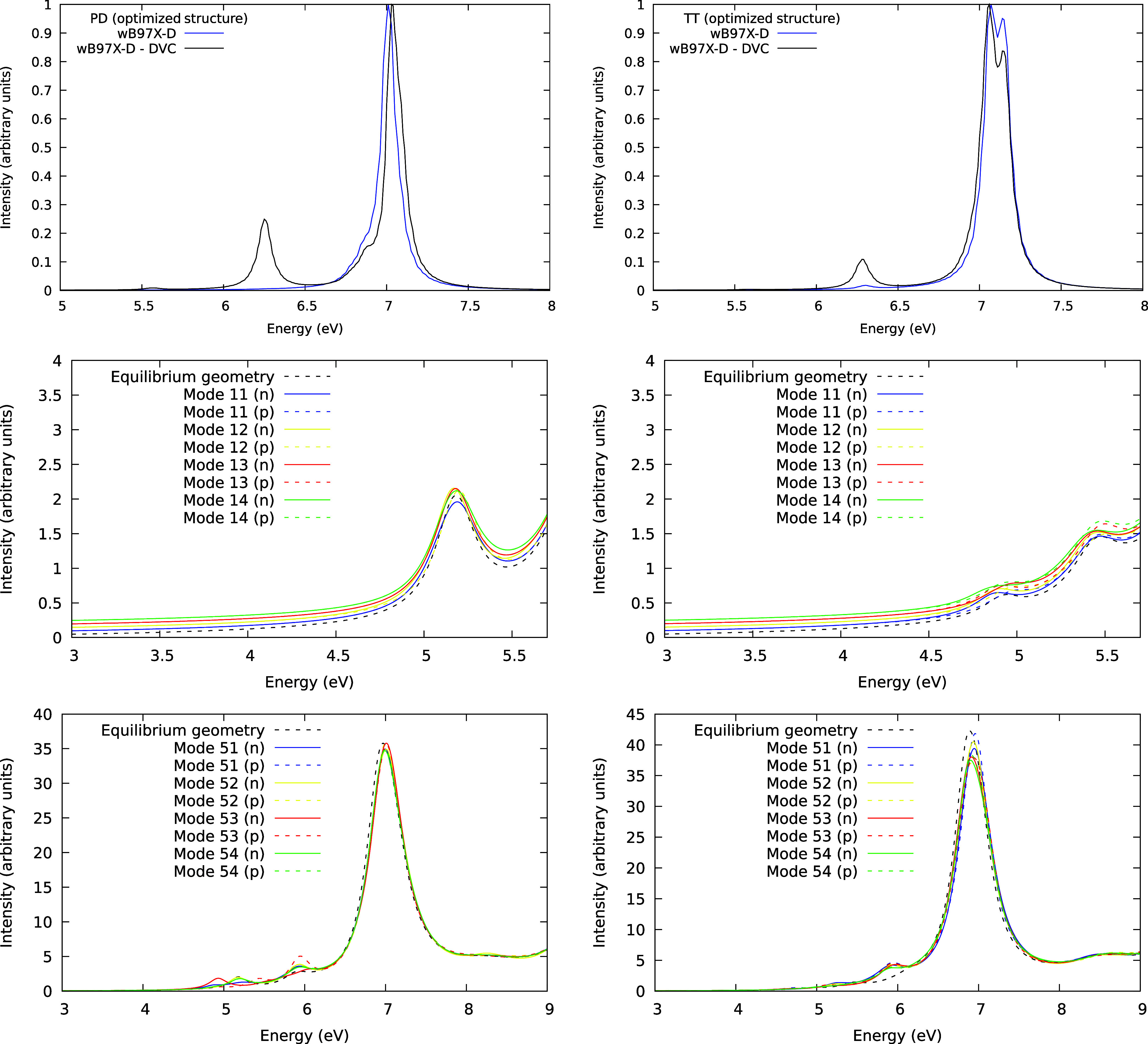
Simulated absorption spectra utilizing the fully optimized structure for the PD dimer (left) and TT dimer (right) using DVC (top panel), compared with the spectra on equilibrium geometry (black lines) and Turbo-Laczos (middle and bottom panels) distorted along inducing modes 11, 12, 13, and 14, (middle panel) and 51, 52, 53, and 54, (bottom panel) and dislocated 10% in both directions along the mode, are also shown. The middle panel spectra are vertically shifted for enhanced visualization of the data. These modes were anticipated to induce an excitation in the same energy range as the ^1^A_1g_ → ^1^B_2u_ and ^1^A_1g_ → ^1^B_1u_, respectively.

For the optimized structures (Figure 6), the DVC method shows an opposite trend for the low-energy band (top panels). In fact, for the TT dimers, even with no distortion (i.e., when we calculate the equilibrium geometry spectrum), a prominent band arises at 5 eV compared to the absence of any transition in the vertical spectra of the monomer equilibrium geometry spectrum (Figure 3). This band indicates that the dimer lowers symmetry compared to the monomer, and its intermolecular interactions are sufficient to allow this transition to happen (which corresponds to the ^1^A_1g_ → ^1^B_2u_ transition in the monomer). The PD dimer, although quite unnoticeable on the vertical spectrum, exhibits a greater increase in intensity on this low-energy band. This could be attributed to the symmetry gained when the structure is indeed on the minima of the PES, which may not necessarily occur on the experimental structure, as it is merely an average of positions. For the energy range between 7.0 and 7.5 eV, the same two bands appear on both dimers but with different intensities for the first band. The TT dimer exhibits the highest intensity, suggesting once again that the break of symmetry imposed by the T shape is the most significant factor for the electronic transitions.

As for the Liouville-Lanczos method, we obtained two spectra for each inducing mode (with positive and negative distortions along the mode). We used the same procedure employed previously to choose the inducing modes. The spectra for the fully optimized PD and TT geometries are shown in Figure 6 (middle and bottom panels), emphasizing the energy region of the first and second forbidden transitions in the monomer, respectively. For the fully optimized TT dimer, the transition splits into two bands at 4.9 and 5.4 eV. The first band for both PD and TT dimers is only slightly affected by the distortions.

In Figure 6 (bottom left panel), it can be seen that the fully optimized PD dimer has its transition at 6 eV, corresponding to the ^1^A_1g_ → ^1^B_1u_ in the monomer, heavily increased by a positive distortion in the inducing mode 53. Interestingly, a negative distortion in the same mode leads to a minimum change in the band intensity compared to the vertical spectrum. The remaining modes increase moderately the OOS. It can also be seen that some bands arise around 5 eV due to the symmetry breaking of the dimer. In Figure 6 (bottom right panel), a distortion in all of the selected modes led to a decrease in the band intensity of the allowed transition (^1^A_1g_ → ^1^E_1u_) for the fully optimized TT dimer, unlike the observed for the fully optimized PD dimer. Surprisingly, there is a strong dependence on the vibronic coupling for the occurrence of a band in the same energy region of the ^1^A_1g_ → ^1^B_1u_ transition in the monomer since all modes lead this band to increase the intensity at 6 eV compared to the equilibrium geometry spectrum.

Overall, both methods yield a satisfactory agreement, though with certain discrepancies. The primary source of these discrepancies lies in the vibrational structure of the bands. Specifically, the optimized structure exhibits two bands between 7.0 and 7.5, whereas the turbo-Lanczos method identifies only one. This discrepancy could be attributed to the shifting of one of the bands closer to the other due to vibrational structures. Notably, this phenomenon is not captured by DVC, as one of its approximations involves considering only the vertical energy. Also, the PD experimental band exhibits a degeneracy break with turbo-Lanczos that is absent in DVC, which can be attributed to the same reason.

## 4 Conclusions

In this work, we simulated the absorption spectra of benzene clusters and crystal in the IR and UV–vis regions. The systems under study were the gas-phase monomer, two dimers extracted from the benzene crystal (i.e., experimental PD and TT dimers), two fully optimized gas-phase dimers (optimized PD and TT dimers), and benzene crystal. The IR spectra show that, from the gas phase to crystal (increasing aggregation), the band related to the C–H stretching modes becomes gradually less intense while the bands related to the bending modes become more intense. Also, dimer spectra have more complex band profiles. None of the normal modes related to the most intense IR bands induce vibronic transitions.

Applying the DVC method, we demonstrated that the molecular aggregation has a strong influence both on the first forbidden transition (^1^A_1g_ → ^1^B_2u_) and also impacts the second one (^1^A_1g_ → ^1^B_1u_), increasing the intensity. The same conclusion holds using the Liouville-Lanczos approach. The band of allowed (intense) transition exhibits a low- and high-energy shoulder in the high-pressure regime for the TT and PD dimers (experimental structures), respectively. In the low-pressure regime (optimized structures), a small split is present for the allowed transition of the TT dimer. Such band modifications indicate the spectroscopic signature of dimer formations.

Our results highlighted that the vibronic coupling effects are key factors in describing the electronic spectra of both the monomer and clusters. The intermolecular interactions in the molecular aggregates uniquely modify the electronic spectra of benzene depending on the underlying dimer structure. Furthermore, in our work, we applied an approach to calculate the vibronic spectra of molecular clusters using the vibrational functions of all molecules present in selected molecular aggregates and not only based on the monomer vibrational function, as proposed in the recent literature.^16,17^ Moreover, the magnitudes of the two effects on the photoabsorption spectra are distinct in each forbidden electronic transition analyzed. Finally, to the best of our knowledge, the vibronic coupling was combined with the Liouville-Lanczos approach for the first time in this work.

## Supporting Information Available

Normal modes and vibrational frequencies (PDF)
