# Supplementary material for: Signatures in Vibrational and Vibronic Spectra of Benzene Molecular Clusters
Source: J Phys Chem A. 2025 Apr 9;129(15):3435–44. doi: 10.1021/acs.jpca.4c08700 (PMC12010323; doi:10.1021/acs.jpca.4c08700)
Supplement: Supplementary file 1 — jp4c08700_si_001.pdf [file jp4c08700_si_001.pdf]

Supporting Information: Signatures in Vibrational and Vibronic Spectra of Benzene Molecular Clusters

Ricardo Montserrat,[†,‡] Amanda D. Torres,[*,†] Ricardo R. Oliveira,[*,†] and Alexandre B. Rocha[*,†]

†*Chemistry Institute, Federal University of Rio de Janeiro, Rio de Janeiro, Brazil - 21941-909*

‡*Instituto Federal do Paraná, Umuarama, Paraná, Brasil - 87507-014*

E-mail: dornelaamanda@pos.iq.ufrj.br; rrodrigues.iq@gmail.com; rocha@iq.ufrj.br

Contents

1 Additional data **S-3**

References **S-4**

1 Additional data

Table S1: Normal mode symmetries, calculated frequencies and experimental frequencies of the vibrational modes of benzene obtained with ωB97X-D/aug-cc-pVTZ level of theory. The relative errors are shown in parenthesis (%).

Mode	Symmetry	Frequency (cm^{-1})	Experimental[S1] (cm^{-1})
1	E_{2u}	413.4370	410 ± 6
2	E_{2u}	413.4682	410 ± 6
3	E_{2g}	626.5269	606 ± 6
4	E_{2g}	626.6524	606 ± 6
5	A_{2u}	699.7174	673 ± 3
6	B_{2g}	724.6762	703 ± 30
7	E_{1g}	883.0750	849 ± 6
8	E_{1g}	883.1272	849 ± 6
9	E_{2u}	1010.0276	975 ± 6
10	E_{2u}	1010.0311	975 ± 6
11	B_{1u}	1029.1249	1010 ± 6
12	B_{2g}	1033.5351	995 ± 30
13	A_{1g}	1033.8759	992 ± 6
14	E_{1u}	1072.8198	1038 ± 3
15	E_{1u}	1072.8270	1038 ± 3
16	B_{2u}	1176.6720	1150 ± 6
17	E_{2g}	1207.6167	1178 ± 6
18	E_{2g}	1207.6397	1178 ± 6
19	A_{2g}	1338.7053	1326 ± 30
20	B_{2u}	1390.0120	1310 ± 6
21	E_{1u}	1528.1504	1486 ± 3
22	E_{1u}	1528.2012	1486 ± 3
23	E_{2g}	1668.2201	1596 ± 30
24	E_{2g}	1668.2338	1596 ± 30
25	B_{1u}	3184.5573	3068 ± 6
26	E_{2g}	3194.6965	3047 ± 6
27	E_{2g}	3194.7084	3047 ± 6
28	E_{1u}	3210.9771	3063 ± 30
29	E_{1u}	3210.9795	3063 ± 30
30	A_{1g}	3221.5685	3062 ± 6

References

(S1) of Standards, N. I.; Technology Experimental data for C_6H_6 (Benzene). 2022; https://cccbdb.nist.gov/exp2x.asp?casno=71432&charge=0.
